# Process evaluation design in a cluster randomised controlled childhood obesity prevention trial: the WAVES study

**DOI:** 10.1186/s12966-014-0112-1

**Published:** 2014-09-10

**Authors:** Tania L Griffin, Miranda J Pallan, Joanne L Clarke, Emma R Lancashire, Anna Lyon, Jayne M Parry, Peymane Adab, On behalf of the WAVES study trial investigators

**Affiliations:** Public Health Building, University of Birmingham, Birmingham, B15 2TT UK

**Keywords:** Cluster randomised controlled trial, Intervention, Childhood obesity prevention, Process evaluation, Implementation fidelity

## Abstract

**Background:**

The implementation of a complex intervention is heavily influenced by individual context. Variation in implementation and tailoring of the intervention to the particular context will occur, even in a trial setting. It is recognised that in trials, evaluating the process of implementation of a complex intervention is important, yet process evaluation methods are rarely reported. The WAVES study is a cluster randomised controlled trial to evaluate the effectiveness of an obesity prevention intervention programme targeting children aged 6–7 years, delivered by teachers in primary schools across the West Midlands, UK. The intervention promoted activities encouraging physical activity and healthy eating. This paper presents the methods used to assess implementation of the intervention.

**Methods:**

Previous literature was used to identify the dimensions of intervention process and implementation to be assessed, including adherence, exposure, quality of delivery, participant responsiveness, context, and programme differentiation.

**Results:**

Multiple methods and tools were developed to capture information on all these dimensions. These included observations, logbooks, qualitative evaluation, questionnaires and research team reflection.

**Discussion:**

Data collection posed several challenges, predominantly when relying on teachers to complete paperwork, which they saw as burdensome on top of their teaching responsibilities. However, the use of multiple methods helped to ensure data on each dimension, where possible, was collected using more than one method. This also allowed for triangulation of the findings when several data sources on any one dimension were available.

**Conclusions:**

We have reported a comprehensive approach to the assessment of the implementation and processes of a complex childhood obesity prevention intervention within a cluster randomised controlled trial. These approaches can be transferred and adapted for use in other complex intervention trials.

**Trial registration number:**

ISRCTN97000586

## Background

There is an increasing body of literature on “complex interventions” in health services research, where the intervention is a programme of interconnected components, which may be delivered or implemented in a variety of ways. The publication of a framework for the development and evaluation of such interventions by the UK Medical Research Council (MRC) [1] has guided recent trials of complex health interventions. One frequent criticism of such trials is the difficulty in explaining the process by which the interventions have had the observed effects and distinguishing the relative contribution of intervention components. To address this there is increasing emphasis on evaluating intervention processes [2,3], including monitoring and reporting the fidelity of intervention implementation (i.e. whether the intervention is delivered as intended) and assessing contextual influences on intervention delivery and outcomes. Such process evaluation will enable us to better understand any inconsistencies between expected and observed outcomes, and help inform transferability and future implementation of the intervention. Despite increasing emphasis on the importance of process evaluation of complex interventions, the literature highlights that the reporting of methods used in trials of complex interventions is still limited and, when undertaken, poorly described [4,5]. Thus there is a need to report the development and implementation of process evaluation methods.

One example of an area of complex intervention research is childhood obesity; this is a growing health problem worldwide, and effective and feasible preventive interventions are urgently required. Such interventions usually include multiple related components, targeting children as well as other stakeholders, and have a wide range of interconnected outcomes. Whilst there have been numerous trials aimed at prevention, summarised in over 30 systematic reviews [6], a major limitation to the interpretation of the trial outcomes is the poor reporting of process measures in existing studies. The most recent Cochrane review of childhood obesity prevention trials [3] found that of 39 trials targeting primary school aged children, around a third did not report any process measures. Those that did, used a variety of approaches, and none included a comprehensive set of process and implementation fidelity assessment tools. The authors concluded that future trials need to include comprehensive process evaluation data in order to interpret outcomes and to guide future implementation [3].

### Approaches to process evaluation

There are several key resources that highlight important aspects for consideration in the development of process evaluation methods for complex intervention trials. However, there is no single defined, agreed comprehensive framework for such evaluation [4]. This is reflected by a lack of uniformity in process evaluation methods presented in the literature [4,7].

One commonly used framework, outlined by Baranowski and Stables [8] and refined by Linnan and Steckler [7], includes seven key dimensions that need to be evaluated: *Context* (environmental aspects of the intervention setting), *Reach* (the proportion of participants who received the intervention), *Fidelity* (whether the intervention is delivered as planned), *Dose delivered and received* (the amount of intervention delivered and the extent to which participants responded to it), *Implementation*, (a composite score of reach, dose and fidelity), and *Recruitment* (methods used to attract participants). Linnan and Steckler emphasise the importance of identifying potential factors that may influence intervention delivery prior to the commencement of the trial rather than working in retrospect. This framework overlaps to some extent with the five dimensions outlined in the RE-AIM framework (Reach, Efficacy, Adoption, Implementation and Maintenance) [9] which is used to assist in the translation of public health intervention research into real world settings. Similarly there is consistency with the taxonomy of implementation outcomes proposed by Proctor et al. [10] (Acceptability, Adoption, Appropriateness, Feasibility, Fidelity, Implementation cost, Penetration and Sustainability).

One key component of process evaluation that is repeatedly highlighted as important is the assessment of fidelity; the extent to which a complex intervention is implemented as intended by the developer [7,11,12]. Fidelity is a component discussed in the process evaluation framework by Linnan and Steckler [7], is stated within Proctor et al’s taxonomy of implementation outcomes and resembles the “implementation” dimension within the RE-AIM framework. However, Dane and Schneider [11] identified it as an independent concept to be monitored during interventions. They used the term ‘implementation fidelity’ and defined five dimensions that should be considered in its assessment: *Adherence*, how well the intervention delivery followed recommended methods; *Exposure*, the amount of intervention received by the participants; *Quality of delivery* of the intervention; *Participant Responsiveness,* how the participants responded to the different intervention components; and *Programme Differentiation*, identifying whether certain aspects of the intervention were more effective than others [11,12].

### Overview of process evaluation methods used in childhood obesity prevention trials

An extensive process evaluation was reported for the PAAC (Physical Activity Across the Curriculum) study in which six evaluation dimensions were considered: Context, Reach, Fidelity, Dose delivered and received and Implementation through a variety of data collection methods (attendance records, surveys, observations, questionnaires and focus groups) [13]. However, although the intervention assessed was school-based, it involved only the single component of incorporating physical activity into classroom-based curricular activities [13]. Two large-scale multicomponent school-based interventions targeting both diet and physical activity; Pathways [14], and the HEALTHY study [15], adopted a range of different assessment methods in their process evaluations. The Pathways trial used structured interviews with teachers, attendance at session observations and, student, teacher and family feedback forms to collect process evaluation data. The HEALTHY trial used observations of intervention sessions, interviews and focus groups (with school staff and children) and teacher feedback forms on class behaviour. Such extensive methods allow for triangulation of data from different sources, however both trials restricted their evaluations to assess only Fidelity, Reach and Dose. A smaller, seven week pilot study (‘Guys Only Activity for Life’), encouraging physical activity amongst 11–13 year old children also restricted their process evaluation to three dimensions, assessing Reach by pupil attendance at intervention sessions, Dose by session observations and heart rate (as a measure of physical activity intensity) and Fidelity by staff surveys and audio taping sessions [16].

Some studies have limited both dimensions assessed and data collection methods in their process evaluations. The 5-a-day Power Plus intervention, to increase fruit and vegetable consumption amongst elementary school children, assessed Participation, Dose and Fidelity using attendance logs, questionnaires and observations [17]. A UK primary school-based intervention study, Project Tomato, promoting fruit and vegetable consumption assessed Implementation and Appreciation in their process evaluation, using only teacher, parent and child questionnaires [18].

Most of the process evaluations from the school-based health promotion intervention literature, a number of which are summarised above, have based their process evaluation on the framework proposed by Linnen and Steckler [7]. However, a number have used alternative approaches. The ‘Switch-Play’ intervention (reducing sedentary behaviour by encouraging physical activity) for 10-year old children [19] focused on the dimensions of implementation fidelity proposed by Dane and Schneider [11]. Three dimensions were assessed: Dose, Quality and Participant Responsiveness, using staff logs and parent/child surveys. The process evaluation of the GLAMA (Girls! Lead! Achieve! Mentor! Activate!) intervention programme [20], which encouraged peer leadership and physical activity amongst girls aged 12–13 and 15–16 years, was directed by the RE-AIM framework. Student feedback, questionnaires and observations were used to evaluate the programme. There are also some health promotion intervention studies which have reported similar methods of data collection in their process evaluations, but have not referred to a specific framework [21,22].

This brief review demonstrates that a variety of process evaluations are reported in the school-based health promotion literature. Recurring limitations include the number of dimensions or nature of the intervention assessed, the data collection methods used or absence of an evaluation framework (or a combination of these limitations).

Whilst there is some overlap between process evaluation frameworks seen in the literature [7,9–11], there are also distinct elements within each framework. Used together, they provide a comprehensive approach to the measurement of processes and fidelity of implementation in complex intervention trials.

In this paper, we add to the current literature by describing a comprehensive approach to process evaluation undertaken in a trial of a complex, primary school-based obesity prevention intervention; the West Midlands ActiVe lifestyle and healthy Eating in School children (WAVES) study. Our intention is not to provide another altogether different approach to process evaluation, but rather present, in detail, the methods used in this evaluation, which has drawn together key aspects of process evaluation previously presented in the health promotion intervention literature. We describe the methods used for evaluating the defined dimensions and discuss the main limitations and challenges faced in undertaking the evaluation. The results of the evaluation will be presented in subsequent papers.

## Methods

### Overview of The WAVES study

The WAVES study is a cluster randomised controlled trial funded by the National Institute for Health Research, Health Technology Assessment Programme. Ethical approval was obtained from the National Research Ethics Service Committee West Midlands, The Black Country (10/H1202/69, 25th November 2010). Full details of the WAVES study and intervention programme will be described elsewhere and are also presented on the study website [23]. In summary, the trial is testing the clinical and cost-effectiveness of an obesity prevention intervention programme, informed by developmental work (Birmingham Healthy Eating & Active lifestyle for Children study, [24]) and designed for children aged 6–7 years (Year 2). A total of 54 schools in the West Midlands (UK) are involved, 26 of which were randomly assigned to the intervention arm. The primary outcome measure of the trial (BMI standard deviation score (BMI z-score)) is assessed only in children consented to participate in the measurement element of the study. However, all children in Year 2 in the intervention schools, regardless of their measurement consent status, were exposed to the intervention activities, which were delivered through schools.

The WAVES study is aiming to test the effectiveness of the intervention in a real life setting, therefore the intervention was designed to be delivered in schools by nominated staff (primarily Year 2 teachers), and external organisations where relevant. Whilst the components within the intervention programme were predefined, it was acknowledged that each school would have different contextual and environmental influences on intervention delivery and response. Thus, from the outset teachers were asked to follow a protocol to ensure key intervention elements were delivered, but adaptation of the resources to meet the needs of their class was also encouraged to maximise the likelihood of full implementation [12,25,26]. This flexibility to accommodate individual school circumstances added complexity to delivery of the intervention and emphasises the importance of evaluating the processes of intervention delivery.

### The WAVES study intervention programme

The WAVES study intervention is a multifaceted 12 month programme run throughout one school year. It is comprised of four key components that relate to the promotion of physical activity and healthy eating, as well as a termly newsletter to reinforce the messages delivered through the various components. Each individual component is briefly described below.

#### Increasing children’s daily physical activity during the school day

Teachers were asked to incorporate moderate to vigorous physical activity (MVPA) into the school day in addition to curricular physical education, with a target of achieving an extra 30 minutes every day. To help schools achieve this target they were provided with a choice of four commercially available physical activity packages (‘Activate’ [27], ‘Positive Play’ [28] , ‘Take 10’ [29] or ‘Wake Up Shake Up’ [30]). Each activity package included a manual and/or a DVD to facilitate delivery of MVPA. The activities did not require children to change into sportswear and could be undertaken in the classroom (except for Positive Play which involved organised playground activities). Activity programmes, similar to those offered by the WAVES study, have been shown to have a positive impact on physical activity levels of children when used in the classroom [31,32].

#### Nutrition education and healthy food preparation skills – ‘cooking workshops’

School staff were asked to deliver three ‘cooking workshops’ to children and their parents/carers throughout the intervention year. The workshops were developed by nutritionists from the WAVES study research team and aimed to improve nutrition knowledge and food preparation skills of children and their families. School staff were trained to deliver the workshops and were provided with materials and resources to enable them to run the workshops in school. Each workshop followed the same structure, three short lessons (10 minutes each) to be delivered in class time prior to the main ‘workshop’ (60–90 minutes), to which parents were invited to attend. Activities included education, interactive puzzles, and making a healthy breakfast, lunch and evening meal.

#### Villa vitality

A programme run at Aston Villa Football Club (AVFC, an English Premier League football/soccer club), focusing on promoting healthy lifestyles in the engaging setting of a prominent sports club. It involved two day trips to AVFC (6 weeks apart) and a football coaching session delivered at school (in week 3) by an AVFC coach. During the AVFC visits children participated in a range of interactive sessions targeting diet and physical activity behaviours. During the intervening six week period, teachers were asked to work on a project with their class and encourage children to undertake weekly health challenges (achieve 60 minutes of activity every day, swap a snack, drink more water, eat a healthy breakfast every day, eat 5 portions of fruit and veg every day and cook a healthy family meal).

#### Signposting

Twice in the intervention year (immediately before the summer holidays when the children were leaving Year 1 and during the Autumn term of Year 2) children were asked to take home information sheets outlining ideas on how to be active and signposting them and their family to local facilities providing physical activity opportunities.

### Development of process evaluation data collection methods

First, the dimensions to be included in the process evaluation were defined, guided by Linnan and Steckler's and Dane and Schneider's frameworks [7,11]. Specific questions were developed and mapped onto the different evaluation dimensions (Table 1). Potential data collection methods were then identified from the literature [12,13,33–35] and through seeking advice from researchers with experience in the field. Tools that are commonly used for process evaluation fall broadly into four groups: 1) checklists or logbooks completed by intervention providers; 2) surveys, interviews or focus groups with participants and intervention providers; 3) behavioural observations by researchers; and 4) use of administrative data, such as attendance or case records. Tools from across these four groups were selected to ensure that the resulting data would address the specific questions and therefore the defined evaluation dimensions. Where possible multiple methods were used to measure the same evaluation dimension in order to triangulate findings and to try to ensure that data were available for each dimension, even if some methods of data collection remained incomplete. We describe the detail of each data collection method developed in the results section below.

**Table 1:** **Summary of research questions assessed in the WAVES study intervention process evaluation**

**Research question**	**Process evaluation dimensions**	**Data source**
Is the intervention being delivered in the way it was intended?	Fidelity^1^/Adherence^2^	Observations
		Logbooks
		Qualitative evaluation
		Questionnaires
How much exposure are children and families getting to each intervention component?	Reach^1^/Dose delivered^1^/Dose recieved^1^/Exposure^2^	Observations
		Logbooks
		Qualitative evaluation
		Questionnaires
What methods are used for encouraging participation in intervention activities?	Recruitment^1^	Observation
		Qualitative evaluation
What quality of intervention is being received?	Quality^2^	Observations
		Logbooks
How well are children and families responding to, and engaging with, the intervention?	Dose received^1^/Participant responsiveness^2^	Observations
		Logbooks
		Qualitative evaluation
Are there intervention components which are more essential than others?	Programme differentiation^2^	Observations
		Logbooks
		Qualitative evaluation
		Questionnaires
Are there contextual and environmental factors which have the potential to influence delivery?	Context^1^	Observations
		Qualitative evaluation
		Questionnaires
		Research team reflection

^1^Based on Process evaluation components outlined by Baranowski and Stables [8] & Linnan and Steckler [7].

^2^Based on implementation fidelity components outlined by Dane and Schneider [11].

## Results

A pragmatic approach to data collection was adopted to maximise response whilst minimising the impact on intervention delivery and the workload for school staff. Data collection methods and tools (teacher logbooks, questionnaires and observation checklists) were designed to make them succinct, and as easy to understand and complete as possible. Researcher completed observation checklists were piloted and tested for inter-rater reliability during the initial implementation phase of the intervention (September – October 2011). Checklists were completed independently by two researchers following observation of the same session. Where differences arose on questions requiring a subjective response (e.g. Quality of delivery), a discussion took place to clarify what was expected for different quality ratings and explanatory text added to the checklist to improve future consistency. This process was repeated during the initial stages of the intervention implementation until high inter-rater reliability was achieved, and then at least once per term to ensure consistency was maintained.

**Figure 1:**
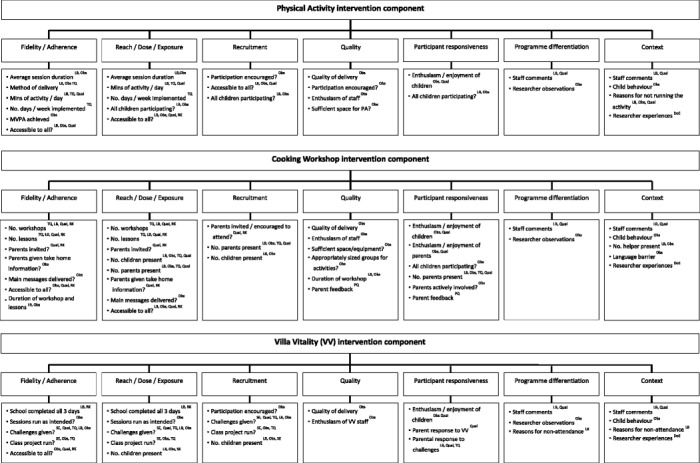
**Sources of information used to inform the WAVES study intervention process evaluation.** DoE: Diary of Experiences. LB: Logbooks. MVPA: Moderate to Vigorous Physical Activity. Obs: Observation checklist. PA: Physical Activity. PE: Parent Evaluation. Qual: Qualitative. SE: Staff Evaluation. RK: Researcher Knowledge. TQ: Teacher questionnaire.

Prior to distribution to teachers, logbooks and questionnaires were reviewed and assessed for face and construct validity by the wider WAVES study research team. The data collected from each information source, and how this relates to the dimensions of process evaluation and fidelity for each intervention component are presented in Figure 1.

### Logbooks

Logbooks were designed to collect self-reported data about intervention delivery from school staff. Separate logbooks were developed for the Physical Activity (PA) and Cooking Workshop (CW) components, which were sent out each term, and the Villa Vitality (VV) component of the intervention, which was sent prior to the 6 week VV programme. The logbooks collected information on the fidelity dimensions: *adherence*; *exposure; quality of delivery*; *participant responsiveness*; and to some extent, *programme differentiation*. The information collected by each logbook together with the fidelity dimensions assessed is presented in detail in Table 2, and summarised in Figure 1. The importance of honest reporting in the logbooks was emphasised to teachers.

**Table 2:** **Content summary of teacher completed logbooks used for the WAVES study intervention process evaluation**

	**Fidelity/Adherence**	**Reach/Dose/Exposure**	**Recruitment**	**Quality**	**Participant responsiveness**	**Programme differentiation**	**Context**
**Physical activity logbook (completed daily)**							
Time of activity	✓	✓					
Duration of activity	✓	✓		✓	✓		
Reason for non-completion							✓
Number of children who did not participate		✓					
Additional comments	✓	✓	✓	✓	✓	✓	✓
**Cooking workshop logbook (1/workshop)**							
***Cooking workshop lessons***							
Number of lessons delivered prior to the workshop?	✓	✓		✓			
Time spent delivering the lessons	✓			✓			
Additional comments	✓	✓	✓	✓	✓	✓	✓
***Cooking workshop***							
Time spent delivering the workshop	✓			✓			
Number of children who did not participate and reasons		✓			✓		
Number of children with a parent/carer present		✓	✓		✓		
Number of helpers present							✓
Additional comments	✓	✓	✓	✓	✓	✓	✓
**Villa vitality logbook**							
***Villa vitality days 1, 2 and 3***							
Number of children attending the day		✓					
Reasons for non-attendance						✓	✓
***Villa vitality project and weekly challenges***							
Time spent delivering the project/challenges	✓			✓			
Number of children who completed each challenge					✓		
Additional comments	✓	✓	✓	✓	✓	✓	✓

School staff were asked to return the logbooks at the end of each term, with two reminders being sent to non-responders. The logbooks were developed and reviewed carefully to try to make them as simple and concise as possible whilst capturing the desired information. Returned logbooks were checked for completeness, and incorrect completion or failure to complete elements of the logbook was fed back to teachers to try to ensure more complete reporting subsequently. At no point was the content of the logbooks used to give feedback to teachers on the quality of intervention delivery.

### Observations

In each intervention school a member of the research team observed the daily physical activity intervention component once per term, at least one of the three cooking workshops and one of the three Villa Vitality sessions during the intervention year. Observation checklists were developed for each of the intervention components and covered a wide range of questions to collect data on the fidelity dimensions: *adherence*; *exposure*; *quality; participant responsiveness*. They also helped to identify any potential contextual factors influencing delivery of the intervention components. The information collected by the checklists and the dimensions assessed are shown in detail in Table 3, and summarised in Figure 1.

**Table 3:** **Content summary of researcher completed observation checklists used for the WAVES study intervention process evaluation**

	**Fidelity/Adherence**	**Reach/Dose/Exposure**	**Recruitment**	**Quality**	**Participant responsiveness**	**Programme differentiation**	**Context**
**Activity** observed ^PA^ ^CW^ ^VV^							✓
**Duration** of activity ^PA^ ^CW^ ^VV^	✓	✓					
Method of delivery ^PA^	✓						
**Number** of **children** ^PA^ ^CW^ ^VV^		✓			✓		
**Number** of **parents** ^CW^		✓			✓		
**Number** of **staff** present, number joining in, and if not why ^PA^ ^CW VV^				✓			
**Number** of **children** present but **not participating** and **why** ^PA^ ^VV^		✓			✓		
Does the leader remind the children of the benefits of the activity? ^PA^			✓	✓			
Does the leader encourage the children to move energetically? ^PA^			✓	✓			
Does the leader encourage the children to participate? ^VV^			✓	✓			
How **enthusiastic** is the teacher? ^PA^ ^CW^ ^VV^				✓			
Do the children have sufficient space? ^PA^				✓			
Overall **quality** of delivery ^PA^ ^CW^ ^VV^				✓			
**Proportion** of children achieving moderate to vigorous activity ^PA^	✓						
**Proportion** of **children enthusiastic** about **/ enjoying** session ^PA^ ^CW^ ^VV^					✓		
**Proportion** of **children** getting **actively involved** in session ^CW^					✓		
**Proportion** of **parents enthusiastic** about **/ enjoying** session ^CW^					✓		
**Proportion** of **parents** getting actively involved in session ^CW^					✓		
Was all of the recommended session **content delivered**? ^CW^ ^VV^	✓	✓					
Children with special educational needs included? ^PA, CW, VV^	✓	✓					
Number of children being **disruptive** ^PA^ ^CW^ ^VV^							✓
Are most children able to follow the instructions given? ^VV^	✓						
Did language appear to be a barrier for parents? ^CW^				✓	✓		✓

PA – Physical activity observation checklist, ^CW^– Cooking workshop observation checklist, ^VV^– Villa Vitality observation checklist.

Researchers were trained to conduct the observations and were familiar with the information requested on the checklists. This helped to minimise the impact of the researcher’s presence during visits as it allowed them to join in the activity (if appropriate) and complete the relevant checklist as soon as possible upon leaving the school. Many of the items on the observation checklists required completion of a five-point likert scale. In an attempt to minimise variation, examples were given to help with interpretation of the scales. However, due to the subjective nature of the checklists, inter-rater reliability assessment continued beyond the piloting of the checklist whereby, on several occasions, two researchers observed the same activity and independently completed the observation checklist. The checklists were then compared for discrepancies. Only occasional minor differences were observed and these were discussed to reach a score allocation consensus.

The observation data were not reviewed during intervention delivery. This was to ensure, where possible, that researchers completing the observations were not influenced by previous observations of intervention delivery in each school. No feedback was given to teachers regarding the quality of intervention delivery following the observations.

### Qualitative evaluation

During the final term of the intervention period qualitative work was undertaken to complement the predominantly quantitative data collected through logbooks and observations. Interviews were conducted with teachers and separate parent and child focus groups run, to explore their respective experiences of the intervention programme. The qualitative evaluation aimed to collect information on the fidelity components: *adherence*; *participant responsiveness*; *programme differentiation*. Parents’ experiences also helped to ascertain the *schools’ adherence* to the programme, identifying whether the requested level of parental involvement in the intervention programme was attempted.

Focus group discussions were chosen as the method of data collection with parents and children as it was hoped they would encourage open expressions of experiences and attitudes [36]. It was also anticipated that children would be more comfortable with the focus group environment as they would gain confidence and support from their peer group [37]. Interviews with staff were chosen for practical reasons and to ensure teachers’ experiences which were unique to their school were captured. Purposive sampling was used to ensure involvement of a range of intervention schools, in terms of geographic location, ethnic demographics, school size, and level of deprivation (based on free school meal entitlement).

Topic guides were developed for each participant group, focussing initially on views of the overall intervention programme, and then more specifically on each component. Participants were invited to express their experiences of the WAVES study and prompts were used where necessary to encourage participants to explain their opinions. The information sought from the qualitative evaluation used to inform the process evaluation is shown in Figure 1. The data will be analysed using the framework approach [38].

### Evaluation questionnaires and researcher experiences

Questionnaires were developed for completion by school staff and parents. Parents/carers who attended the cooking workshops and/or VV were asked to complete evaluation questionnaires, whilst school staff were asked to complete a questionnaire in relation to VV as well as a questionnaire covering all aspects of the WAVES study intervention programme. Data on the wider contextual influences on intervention delivery were collected through a questionnaire completed by the head or deputy head teacher. This questionnaire asked for information on the school’s current activities in terms of nutrition, physical activity and any other aspects of the school environment that were perceived to have an important influence on intervention delivery.

Reflective diaries, completed by the research team for each school, were used to supplement the assessment of the contextual influences on intervention delivery (primarily ascertained through the observation checklists and the head teacher questionnaires). Following each school visit researchers recorded their overall experience of the school, including their impressions of the school ethos and any key points they felt may be relevant to the implementation of the WAVES study intervention.

## Discussion

We have presented the approach and methods used to comprehensively evaluate process and implementation fidelity in relation to delivery of a complex intervention as part of a cluster randomised controlled trial of a childhood obesity prevention programme (the WAVES study). We have outlined the design of a variety of methods and how these can be used in combination to assess the different process evaluation dimensions.

Many previous childhood obesity prevention trials have not undertaken process evaluations, and those which have reported assessing intervention processes, have focused on a limited set of assessments. Aspects most commonly assessed are: intervention adherence (number of sessions delivered), exposure (numbers attending or participating), dose or intensity of intervention, and programme satisfaction among participants or those delivering the intervention [3]. The inclusion of a wider range of assessments in this study will allow us to combine evaluation of process with trial outcomes [1], and test the theoretical pathways of change leading to any observed effects and thus aid the interpretation of the findings. By examining processes related to each individual intervention component, we will have some indication of which components were most influential in terms of any effect observed. The data on fidelity of intervention delivery will enable us to develop a measure of how well the intervention programme was implemented in each school, and thus analyse trial outcomes in relation to the degree of fidelity achieved. Furthermore, insights gained from the wider process evaluation, particularly from the qualitative exploration of contextual influences on intervention delivery and response will enable us to assess the impact of context on trial outcomes. In addition, the data will also inform wider implementation of the intervention beyond the study setting.

There are limitations to our approach, and a number of practical challenges that we came across in undertaking the range of methods described. We acknowledge that it was not possible to fully assess validity and reliability of the logbooks, checklists and questionnaires used as there is no current gold standard for fidelity assessment, and many existing tools are intervention specific. However as previously described, where possible, tools were piloted and inter-reliability tests conducted. A major challenge and limitation to the methods used was the additional workload that the evaluation created for teachers who were delivering the intervention, particularly in relation to the logbooks. Therefore completion of logbooks was at times erratic, and data from some schools were incomplete. Anecdotally, it appeared that some teachers completed the logbooks in blocks of time, rather than daily, which may have also introduced recall error.

Whilst teachers were encouraged to report all information honestly in the logbooks and questionnaires, and an explanation was provided of why this was so critical, it is still possible that they recorded what they thought they should be delivering rather than the reality of what actually happened (social desirability bias). Data triangulation will enable us to explore potential bias in reporting, for example observation data will be compared with the data obtained from the teachers’ logbooks.

For the observations, the initial plan was for researchers to visit schools unannounced. However, this proved impractical as there were often changes to school schedules resulting in failed observations. Observation visits were therefore pre-arranged with schools, which meant schools had prior knowledge that the session would be observed, potentially influencing the delivery of the session. However, in practice this did not appear to happen as it became clear through the process of observation whether or not the children were familiar with the activities and the checklist design allowed the researchers to report their interpretation of this. As the trial is assessing effectiveness of an intervention in a ‘real world’ setting, the process evaluation was designed to avoid influencing delivery of the intervention. However, during observations researchers noted that some school staff perceived these as a source of support for intervention delivery. This posed a challenge to the researchers, however they had been instructed in advance not to offer any specific feedback and so any influence on intervention delivery during the observations was minimised.

Recruitment of participants for the qualitative studies proved a challenge. Once again, this placed an additional claim on teachers’ time. Also, the data collection period coincided with national assessments resulting in a busy time of year for teachers and children in Year 2. This was compounded by the London Olympics and the Queen’s Golden Jubilee in the summer term 2012, resulting in additional activities further calling on teachers’, pupils’ and parents’ time during the qualitative data collection period.

Where possible, we captured data through a variety of methods so that we could triangulate findings. If data from two different sources provide conflicting information, a judgement will need to be made regarding which source is more reliable. The comprehensive nature of the process evaluation poses a major challenge in terms of the amount and range of data collected. Analyses of the data will be guided by the dimensions of evaluation and the questions mapped to these dimensions (Table 1). However, in practice there is a huge quantity of different types of data, each with specific issues that need addressing within the data cleaning and analysis process. The research team are currently in the process of developing a detailed plan of analysis to deal with these issues and fulfil the process evaluation objectives.

Whilst much emphasis has been placed on the importance of undertaking process evaluations, there is no definitive guidance on how this should be undertaken. The MRC is currently developing guidance on process evaluation of complex public health intervention trials [39]. However, until such guidance is finalised, there is no set approach. Process evaluation is a broad term encompassing not only a wide range of approaches but also multiple objectives. The MRC guidance on complex intervention evaluation outlined three core aims of process evaluation: assessment of quality and quantity of intervention implementation, clarification of the theoretical pathways through which the intervention will have its intended effect, and assessment of contextual factors influencing the delivery and outcomes of the intervention [1]. In our approach we have addressed these core aims. A particular strength of our approach is that we have employed methods to gather data that will enable us to test and further refine the theoretical pathways of change that underpin the intervention programme. The value of incorporating theory driven approaches into evaluation of complex intervention trials in this way, and the synergy between experimental outcome evaluation and programme theory development and evaluation is increasingly being highlighted [40].

Our approach to process evaluation resonates strongly with Grant et al.’s recently published framework for process evaluation of cluster-randomised controlled trials [4], particularly in terms of reach, adherence, response, context and testing the theoretical pathways that lead to the observed effects. Two further dimensions of process evaluation that are included in the Grant framework are maintenance (sustainability) and potential unintended consequences of the intervention. Although at the outset we did not explicitly intend to assess sustainability and unintended consequences of the intervention programme, our data collection methods, particularly the qualitative studies, will enable us to assess these dimensions in our analysis.

## Conclusion

Although the publication of trial protocols has now become an established norm, the description of the protocol and methods for process evaluation lags behind. The WAVES study is a large scale trial of a multi-faceted intervention programme designed and monitored by researchers but implemented by schools and external organisations. We have described in detail key dimensions of process evaluation and multiple methods to capture the relevant data. Such detail has been lacking in previous literature and cannot be sufficiently described when presenting the results. This paper should serve as a useful resource for those undertaking future process evaluations within trials of complex interventions.
